# Applications of molecular replacement to G protein-coupled receptors

**DOI:** 10.1107/S090744491301322X

**Published:** 2013-10-18

**Authors:** Andrew C. Kruse, Aashish Manglik, Brian K. Kobilka, William I. Weis

**Affiliations:** a Molecular and Cellular Physiology, Stanford University, 279 Campus Drive, Stanford, CA 94305, USA; b Structural Biology, Stanford University, Fairchild Building, Stanford, CA 94305, USA

**Keywords:** molecular replacement, G protein-coupled receptors, GPCRs, membrane proteins

## Abstract

G protein-coupled receptors (GPCRs) are a large class of integral membrane proteins involved in regulating virtually every aspect of human physiology. Despite their profound importance in human health and disease, structural information regarding GPCRs has been extremely limited until recently. With the advent of a variety of new biochemical and crystallographic techniques, the structural biology of GPCRs has advanced rapidly, offering key molecular insights into GPCR activation and signal transduction. To date, almost all GPCR structures have been solved using molecular-replacement techniques. Here, the unique aspects of molecular replacement as applied to individual GPCRs and to signaling complexes of these important proteins are discussed.

## Abstract 2

The use of molecular replacement in solving the structures of G protein-coupled receptors is discussed, with specific examples being described in detail.

## 1. Introduction

Transmembrane signaling plays a critical role in biology, allowing cells to sense and respond to the surrounding environ­ment. In humans, one of the largest classes of transmembrane signaling proteins is the G protein-coupled receptor (GPCR) family, which comprises approximately 800 members (Kroeze *et al.*, 2003 ▶). As essential regulators of almost every aspect of human physiology, GPCRs are among the most important targets in the treatment of disease. Owing to the profound biomedical importance of GPCRs, a high priority has long been placed on elucidation of the GPCR structure and its relationship to receptor function. However, until recently high-resolution structural information was available for only a single GPCR, bovine rhodopsin.

Recent years have seen a rapid expansion of GPCR structural biology following the report of the structure of the human β_2_ adrenergic receptor in 2007 (Rasmussen *et al.*, 2007 ▶; Cherezov *et al.*, 2007 ▶). Crystal structures of 20 unique GPCRs have been published, including nine structures in 2012 alone (Venkatakrishnan *et al.*, 2013 ▶). In addition to the increasing diversity of GPCRs for which structures have been determined, two of them, rhodopsin and the β_2_ adrenergic receptor (Scheerer *et al.*, 2008 ▶; Rasmussen, DeVree *et al.*, 2011 ▶), have been solved in fully active conformations, and the structure of the β_2_ adrenergic receptor has been solved in complex with the heterotrimeric G protein Gs. Obtaining these structures has been facilitated by developments in protein purification, engineering, crystallization and microdiffraction X-ray data collection (Smith *et al.*, 2012 ▶).

## 2. Advances in GPCR biochemistry and crystallization

The explosion of GPCR structural biology rests in a large part on advances in biochemical and crystallization methods that should apply to membrane proteins in general. These include the development of highly stabilizing lipid-like detergents (Chae *et al.*, 2010 ▶), lipidic mesophase crystallization (Caffrey & Cherezov, 2009 ▶), stabilizing antibody fragments (Day *et al.*, 2007 ▶) and the use of fusion proteins (Rosenbaum *et al.*, 2007 ▶). These approaches have important implications for molecular-replacement phasing of GPCR crystals, as they influence the type of lattice packing and the contents of the asymmetric unit.

**Figure 1:**
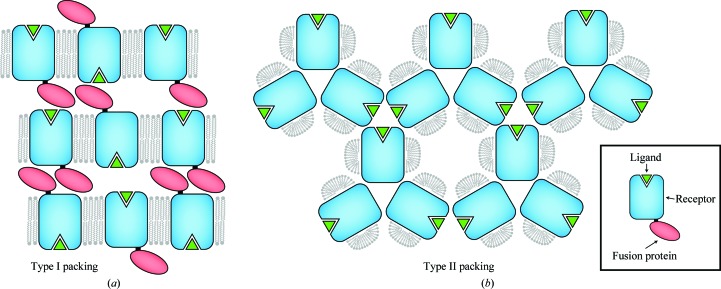
Lattice packing in membrane-protein crystals. Two major types of lattice packing are commonly observed in membrane-protein crystals, termed type I packing (*a*) and type II packing (*b*). Lipidic mesophase methods have yielded exclusively type I packing, allowing the rapid validation of a molecular-replacement search result.

Lipidic mesophase crystallography was first developed in the late 1990s (Landau & Rosenbusch, 1996 ▶), but did not see widespread use until the development of syringe-mixing methods (Caffrey & Cherezov, 2009 ▶) that allowed the straightforward manipulation of the small sample quantities typical for recombinant purified membrane proteins. In all structures reported to date for crystals grown using lipidic mesophase and bicelle methods, a ‘type I’ lattice packing is observed in which the membrane proteins align in parallel stacked layers of alternating lipidic and aqueous phases (Fig. 1 ▶). Thus, one rapid method for validating a questionable molecular-replacement solution is examination of the lattice for type I packing. Mesophase crystallography also has an important implication for structure determination of GPCRs and other membrane proteins, in that the most common crystallization format uses glass plates to enclose the sample. This allows straightforward viewing of the samples, but the difficulty of harvesting and stabilizing crystals grown in this format complicates the usual heavy-atom soaking methods. Nonetheless, hanging-drop vapor diffusion has been successfully used to obtain crystals in lipidic mesophases (Rasmussen, Choi *et al.*, 2011 ▶), and the structure of the seven-transmembrane protein Smoothened has recently been solved by experimental phasing (Wang, Wu *et al.*, 2013 ▶).

**Figure 2:**
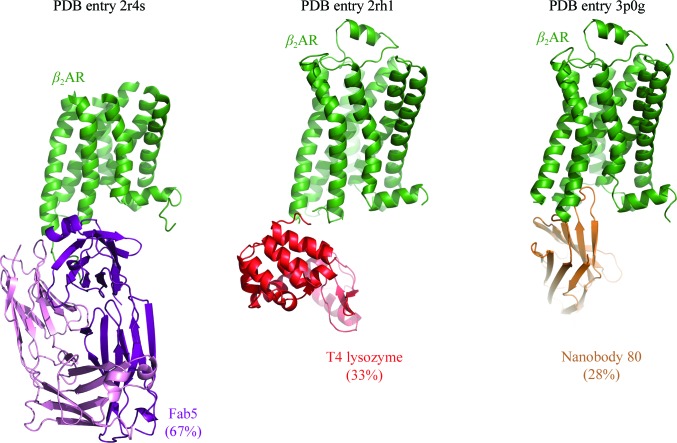
Fusion proteins and chaperones in GPCR crystallography. Extensive use of antibody fragments and fusion proteins has facilitated GPCR structure determination and molecular-replacement phasing. Shown here are structures of β_2_AR solved with the aid of a Fab fragment, T4 lysozyme fusion protein and a camelid nanobody. In each case, the percentage of the final model comprised by the crystallization chaperone is indicated.

One of the most significant advances in GPCR crystallo­graphy is the use of fusion proteins to facilitate the formation of lattice contacts by expanding the otherwise limited hydrophilic surface area of GPCRs. The fusion-protein strategy has been used to solve most of the GPCR structures reported to date (Venkatakrishnan *et al.*, 2013 ▶; Wang, Jiang *et al.*, 2013 ▶; Wacker *et al.*, 2013 ▶; Wang, Wu *et al.*, 2013 ▶). A related approach is the use of antibody fragments, particularly Fab fragments and V_HH_ fragments from camelids, termed ‘nanobodies’. These proteins can bind to GPCRs to form rigid complexes and can stabilize particular conformations to allow the crystallization of specific signaling states for a given receptor. The use of fusion proteins and antibody fragments can greatly facilitate molecular-replacement phasing, as they provide excellent search models and may constitute a large fraction of the asymmetric unit contents (Fig. 2 ▶). The phasing of the first human GPCR structure, that of the β_2_ adrenergic receptor, was accomplished using a cocrystallized Fab fragment as a molecular-replacement search model (Rasmussen *et al.*, 2007 ▶). As we discuss below, for higher resolution structures the presence of a small fusion protein is sufficient for phasing even in the absence of a suitable receptor search model.

## 3. Molecular-replacement phasing of a GPCR signaling-complex structure

**Figure 3:**
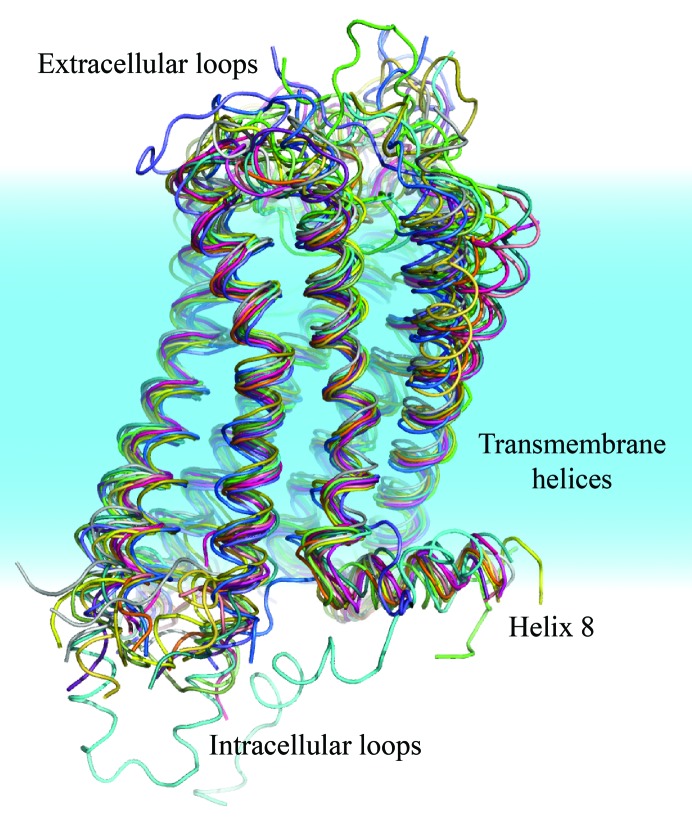
Alignment of GPCR structures. One inactive conformation structure of each unique GPCR published to date was aligned with that of β_2_AR (PDB entry 2rh1; Cherezov *et al.*, 2007 ▶). Structures show exceptional fold conservation, even with pairwise sequence identities that are lower than 25% in some cases.

GPCRs exhibit a remarkable overall conservation of fold, particularly within the transmembrane region, whereas greater structural diversity is observed within the extracellular and intracellular loops (Fig. 3 ▶). Owing to the high degree of structural homology among family A GPCRs, molecular-replacement phasing of these structures is typically relatively straightforward, even in cases with low sequence identity. Indeed, all seven transmembrane receptor structures published to date have been solved by molecular replacement, with the exceptions of bovine rhodopsin, which was the first GPCR structure to be solved (Palczewski *et al.*, 2000 ▶), and the recently determined structure of the Smoothened receptor (Wang, Wu *et al.*, 2013 ▶). Notably, the first structure of the human β_2_ adrenergic receptor (β_2_AR) used phases that were obtained from a Fab fragment rather than rhodopsin as a receptor template for initial structure determination (Rasmussen *et al.*, 2007 ▶). As an example of a challenging GPCR structure solution by molecular replacement, we discuss here the structure of the β_2_ adrenergic receptor in complex with the heterotrimeric G protein Gs (Rasmussen, DeVree *et al.*, 2011 ▶).

The crystallization of the β_2_ adrenergic receptor–Gs signaling complex (β_2_AR–Gs) represents the culmination of years of biochemical work leading to the production of a stable monodisperse complex of the component proteins. The use of a β_2_ adrenergic receptor-­T4 lysozyme fusion protein was essential for crystallization, and strongly diffracting crystals could only be obtained in the presence of the complex-stabilizing nanobody Nb35. These techniques, combined with the use of specialized detergents and lipids, allowed the production of large crystals in lipidic sponge phase which were approximately 25 × 50 × 100 µm in size (Rasmussen, DeVree *et al.*, 2011 ▶). The crystals showed strong diffraction to 3 Å resolution or better in many cases. Data collection involved the use of microdiffraction, and a final data set was compiled using diffraction data from 20 crystals. Solvent-content analysis indicated that the asymmetric unit almost certainly contained a single complex.

To solve the structure of β_2_AR–Gs, molecular-replacement searches were attempted using a variety of component proteins in the complex. The use of the G protein βγ heterodimer (PDB entry 1gp2; Wall *et al.*, 1995 ▶) as the initial search model proved to be most fruitful, yielding a convincing solution in *Phaser* (McCoy *et al.*, 2007 ▶) with a translation-function (TF) *Z*-score of 20. Following this partial solution, the other components could not be convincingly located. Although the G protein α subunit comprised the next largest fraction of the asymmetric unit, molecular-replacement searches were unsuccessful. Moreover, a search for the next largest component, the β_2_AR, also failed. Searching for β_2_AR (PDB entry 3p0g; Rasmussen *et al.*, 2007 ▶) in *Phaser* using the partial solution for Gβγ gave a TF *Z*-score of only 6.1, and comparison with the final structure reveals that the receptor was incorrectly placed.

**Figure 4:**
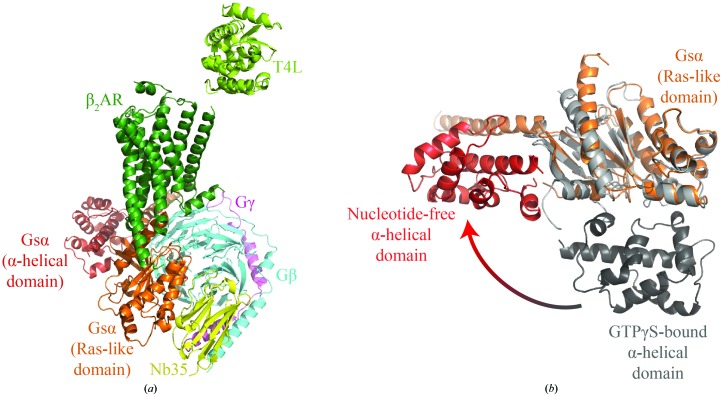
Structure of the β_2_AR–Gs complex. Molecular-replacement phasing was complicated by the large number of components (*a*), as well as the large conformational changes between the nucleotide-bound Gs α-subunit search model and the nucleotide-free signaling complex (*b*).

The G protein α subunit consists of Ras-like and α-helical subdomains. On the basis of this structure and electron-microscopy data (Westfield *et al.*, 2011 ▶) suggesting interdomain flexibility, these two subdomains were used separately in the molecular-replacement search. This strategy yielded a partial solution comprised of the previously placed Gβγ subunit and the Gs α Ras-like domain. Placement of the Ras-like domain was unambiguous, with a TF *Z*-score of 16. Using this partial solution as the basis for subsequent molecular-replacement searches allowed the straightforward location of the remaining components, giving a suitable model for manual building and subsequent refinement (Fig. 4 ▶).

The final structure of the β_2_AR–Gs complex reveals the reasons for the difficulty in initially locating the components of the complex. Principal among these is the remarkable opening of the Gs α subunit, which exhibits an unprecedented 120° rotation of the α-helical domain relative to the Ras-like domain. Several other components are poorly ordered, including T4 lysozyme and the α-helical domain of the G protein. These factors are likely to account for the initial difficulty in locating these components of the complex.

Notably, a search with β_2_AR failed despite a highly similar search model derived from a nanobody-stabilized active-state structure of this receptor (PDB entry 3p0g). Upon refinement, it became clear that the extracellular portions of the receptor did not engage in direct lattice contacts and exhibited high temperature factors relative to the rest of the structure. Owing to the poorly ordered nature of this portion of the receptor, the overall contribution of this molecule to the scattering is likely to be less than would be expected based on size alone, perhaps contributing to the difficulty in locating it early in the molecular-replacement search. In addition, following refinement it became apparent that 12 residues of intracellular loop 3 adopt an ordered α-helical structure contiguous with transmembrane helix 5, a feature that was missing in the initial search model. Nonetheless, following placement of other components the receptor could be easily located with *Phaser*.

## 4. Future of molecular replacement in GPCR structure determination

The structures of GPCRs determined to date have relied overwhelmingly on molecular replacement, which is in a large part enabled by the remarkable structural conservation among family A GPCRs. However, as GPCR structural biology expands, it is likely that the currently available structures will be insufficient for molecular replacement. This is particularly the case for GPCRs from other families and non-G protein-coupled seven-transmembrane helix proteins, which show little or no discernable sequence similarity to family A receptors. However, current GPCR crystallization strategies, such as the use of fusion proteins, may be able to facilitate structure determination even for highly dissimilar receptors without the use of experimental phase information.

To assess this possibility, we sought to solve the structure of the β_2_AR-T4 lysozyme fusion protein using no prior receptor structures. This structure, reported in 2007 (Cherezov *et al.*, 2007 ▶), was the first example of the T4 lysozyme (T4L) fusion strategy. β_2_AR-T4L crystallized in space group *C*2, with a single molecule per asymmetric unit. The receptor construct contained T4L in place of the third intracellular loop of the receptor, and a data set was obtained to 2.4 Å resolution.

To solve this structure without using a receptor search model, we began by performing molecular replacement using the full data set to 2.4 Å resolution with T4L (PDB entry 4lzm; Bell *et al.*, 1991 ▶) as the sole search model. A convincing solution was quickly identified in *Phaser*, with a TF *Z*-score of 7.1. Examination of the lattice packing revealed layers of T4L molecules separated by a suitable spacing to accommodate a lipidic layer containing the receptor seven-transmembrane domain. Since T4L represents 34% the diffracting matter in the asymmetric unit, the partial solution for T4L alone was insufficient to generate useful electron-density maps. To address this, we made use of the prior knowledge that GPCR transmembrane domains are helical. Using a 19-amino-acid polyalanine α-helix as a search model, we performed molecular replacement in *Phaser* starting from the partial solution consisting of T4L alone. A number of plausible solutions were obtained, with the best-scoring solution having a TF *Z*-score of 10.5. In addition, the placement of the helix within the asymmetric unit showed a stacked layer arrangement, which is consistent with a correct solution.

**Figure 5:**
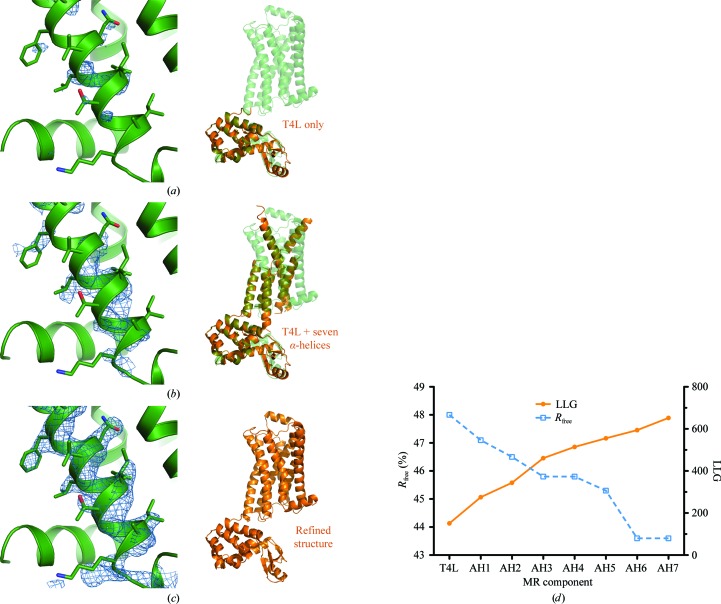
Fragment-based molecular replacement of a GPCR. As a model case, the structure of β_2_AR was solved by molecular replacement using T4 lysozyme followed by helical fragments. (*a*), (*b*) and (*c*) depict 2*F*_o_ − *F*_c_ maps contoured at 1.5σ for transmembrane helix 1, which was not present in the partial MR models, with the final refined structure in green. In (*a*) only T4 lysozyme had been placed; in (*b*) seven α-helical fragments had been added and (*c*) shows a transmembrane helix 1 OMIT map calculated with the final refined structure. In (*d*), the *Phaser* log-likelihood gain (LLG) and *R*_free_ after three cycles of refinement in *phenix.refine* are plotted as a function of the number of fragments placed. AH1 denotes the first α-helix, AH2 the second and so forth.

Proceeding in this manner, six additional transmembrane helices could be located sequentially, as summarized in Table 1 ▶ and Fig. 5 ▶. Notably, the seven helical fragments located by molecular replacement comprised transmembrane helices 2–7, with two of the helical fragments spanning the full length of transmembrane helix 3. Transmembrane helix 1 was un­modeled, but was easily located by inspection of electron-density maps. In each case, the helix was correctly oriented from the N-terminus to the C-terminus. Following the placement of the final transmembrane helix in this manner, inspection of the *F*_o_ − *F*_c_ electron-density map showed clear density for unmodeled features (Fig. 5 ▶), and iterative manual building and refinement were straightforward from this point.

It is likely that the relatively high resolution of this structure facilitated its solution by molecular replacement, so we re­peated the process with additional structures. The first, that of the μ-opioid receptor (μOR) in space group *C*2 at 2.8 Å resolution (Manglik *et al.*, 2012 ▶), was solved in this manner following an identical procedure to that described above. Although the initial placement of T4L gave a low TF *Z*-score, this is common in monoclinic space groups, and the high LLG together with a layered packing typical of LCP crystals indicated that the solution was likely to be correct. Subsequent placement of α-helices starting from this partial solution was easily achieved (Table 2 ▶), although a slight overlap occurred in two helical fragments and was deleted owing to steric clash. A final attempt with a lower resolution structure, that of active β_2_AR bound to a nanobody with data to 3.5 Å resolution (Rasmussen, Choi *et al.*, 2011 ▶), was entirely unsuccessful. An initial search for the nanobody alone using *Phaser* failed to correctly locate the molecule, precluding a subsequent helical fragment search.

Two possible explanations may account for the inability to place the nanobody. The first is that molecular replacement of molecules primarily composed of β-strands is often challenging relative to α-helical and mixed proteins. An alternative explanation is that the lower resolution of the active β_2_AR data set presents challenges for accurate molecular replacement as the rotational orientation of helices about their axis is not well defined in this case. To test this possibility, we followed the procedure for the δ-opioid receptor using data truncated at 3.4 Å resolution. Although the placement of T4 lysozyme was successful, with a TF *Z*-score of 11.7, multiple attempts to place the polyalanine α-helix were unsuccessful, suggesting that the method depends on the use of moderately high-resolution data.

Despite these limitations, a number of GPCR structures have been solved to resolutions of 2.8 Å or better, and the method presented here should be a tractable approach to solving these structures should molecular replacement prove impractical. This is particularly likely to be true of family B and family C GPCRs, as well as other seven-transmembrane receptors such as Frizzled family proteins, Smoothened receptors, PAQR family receptors and other helical membrane proteins for which no suitable molecular-replacement template is currently available. Indeed, the recently reported structure of the Smoothened receptor, a GPCR homolog, was solved by experimental phasing because no GPCR structure was suitable as a molecular-replacement template (Wang, Wu *et al.*, 2013 ▶). In principle, soluble crystallization-chaperone structures and helical search models could be used to solve the structure of any strongly diffracting helical membrane protein that might be crystallized either as a fusion protein or in complex with an antibody fragment, or which contains a soluble domain of known structure (*i.e.* Frizzled proteins, family B GPCRs and family C GPCRs).

## Figures and Tables

**Table 1:** Fragment-based molecular-replacement solution of the β_2_AR-T4L fusion Molecular replacement was performed in *Phaser* using helical fragments following the initial placement of T4 lysozyme. A gradual fall in *R*_free_ is observed, with a concomitant improvement in map quality (Fig. 5 ▶).

Search model	TF *Z*-score	LLG	*R*_free_ (after three cycles) (%)
T4 lysozyme	7.1	151	48.0
α-Helix 1	10.1	275	47.1
α-Helix 2	10.5	344	46.5
α-Helix 3	10.1	461	45.8
α-Helix 4	9.8	514	45.8
α-Helix 5	8.6	555	45.3
α-Helix 6	8.2	595	43.6
α-Helix 7	9.1	652	43.6

**Table 2:** Fragment-based molecular-replacement solution of μOR-T4L fusion Molecular replacement was performed in *Phaser* using helical fragments following the initial placement of T4 lysozyme, analogous to Table 1 ▶.

Search model	TF *Z*-score	LLG	*R*_free_ (after three cycles) (%)
T4 lysozyme	4.1	178	50.4
α-Helix 1	8.9	252	47.6
α-Helix 2	10.0	332	47.6
α-Helix 3	9.3	413	47.9
α-Helix 4	10.6	493	48.0
α-Helix 5	8.8	552	49.0
α-Helix 6	9.5	624	47.7
α-Helix 7	8.6	696	46.7
